# Hypertrophic cardiomyopathy is characterized by alterations of the mitochondrial calcium uniporter complex proteins: insights from patients with aortic valve stenosis versus hypertrophic obstructive cardiomyopathy

**DOI:** 10.3389/fphar.2023.1264216

**Published:** 2023-11-22

**Authors:** Vera Paar, Michael Haslinger, Philipp Krombholz-Reindl, Stefan Pittner, Matthias Neuner, Peter Jirak, Tobias Kolbitsch, Bernd Minnich, Falk Schrödl, Alexandra Kaser-Eichberger, Kristen Kopp, Andreas Koller, Clemens Steinwender, Michael Lichtenauer, Fabio C. Monticelli, Rainald Seitelberger, Uta C. Hoppe, Christian Dinges, Lukas J. Motloch

**Affiliations:** ^1^ Department of Internal Medicine II, Paracelsus Medical University, Salzburg, Austria; ^2^ Department of Cardiac Surgery, Paracelsus Medical University, Salzburg, Austria; ^3^ Department of Legal Medicine and Forensic Psychiatry, Paris-Lodron University of Salzburg, Salzburg, Austria; ^4^ Department of Anesthesiology, Perioperative Medicine and Intensive Care Medicine, Paracelsus Medical University, Salzburg, Austria; ^5^ Department of Internal Medicine, Hospital Gmünd, Lower Austria, Austria; ^6^ Department of Environment and Biodiversity, Paris-Lodron University of Salzburg, Salzburg, Austria; ^7^ Center for Anatomy and Cell Biology, Institute of Anatomy and Cell Biology Salzburg, Paracelsus Medical University, Salzburg, Austria; ^8^ Research Program for Experimental Ophthalmology and Glaucoma Research, Department of Ophthalmology and Optometry, Paracelsus Medical University, Salzburg, Austria; ^9^ Department of Cardiology, Kepler University Hospital, Medical Faculty, Johannes Kepler University Linz, Linz, Austria; ^10^ Department of Internal Medicine II, Salzkammergut Klinikum, OÖG, Vöcklabruck, Austria

**Keywords:** left ventricular hypertrophy, aortic valve stenosis, hypertrophic obstructive cardiomyopathy, fibrosis, calcium, mitochondria

## Abstract

**Introduction:** Hypertrophies of the cardiac septum are caused either by aortic valve stenosis (AVS) or by congenital hypertrophic obstructive cardiomyopathy (HOCM). As they induce cardiac remodeling, these cardiac pathologies may promote an arrhythmogenic substrate with associated malignant ventricular arrhythmias and may lead to heart failure. While altered calcium (Ca^2+^) handling seems to be a key player in the pathogenesis, the role of mitochondrial calcium handling was not investigated in these patients to date.

**Methods:** To investigate this issue, cardiac septal samples were collected from patients undergoing myectomy during cardiac surgery for excessive septal hypertrophy and/or aortic valve replacement, caused by AVS and HOCM. Septal specimens were matched with cardiac tissue obtained from post-mortem controls without cardiac diseases (Ctrl).

**Results and discussion:** Patient characteristics and most of the echocardiographic parameters did not differ between AVS and HOCM. Most notably, the interventricular septum thickness, diastolic (IVSd), was the greatest in HOCM patients. Histological and molecular analyses showed a trend towards higher fibrotic burden in both pathologies, when compared to Ctrl. Most notably, the mitochondrial Ca^2+^ uniporter (MCU) complex associated proteins were altered in both pathologies of left ventricular hypertrophy (LVH). On the one hand, the expression pattern of the MCU complex subunits MCU and MICU1 were shown to be markedly increased, especially in AVS. On the other hand, PRMT-1, UCP-2, and UCP-3 declined with hypertrophy. These conditions were associated with an increase in the expression patterns of the Ca^2+^ uptaking ion channel SERCA2a in AVS (*p* = 0.0013), though not in HOCM, compared to healthy tissue. Our data obtained from human specimen from AVS or HOCM indicates major alterations in the expression of the mitochondrial calcium uniporter complex and associated proteins. Thus, in cardiac septal hypertrophies, besides modifications of cytosolic calcium handling, impaired mitochondrial uptake might be a key player in disease progression.

## 1 Introduction

Cardiovascular diseases resulting in lethal arrhythmias and sudden cardiac death constitute one of the most common causes of death worldwide (Kumar et al., 2021). As young individuals without any preliminary symptoms are also frequently affected, they therefore represent a crucial health problem. Independent from symptoms and etiology, left ventricular hypertrophy (LVH) has been found to act as a strong independent risk factor for sudden cardiac death (Haider et al., 1998; Aro et al., 2017; Stewart et al., 2018). Generally, LVH may be caused by various factors, such as physiologic adaptions in athletes, or may be the result of genetic aberrations, as well as a compensatory mechanism in response to left ventricular outflow tract (LVOT) obstruction. Even concentric remodelling, the precursor of concentric and eccentric LVH, shows an increased risk for heart failure and sudden cardiac death (Aro et al., 2017), mainly through the generation of lethal (ventricular) arrhythmias.

Cardiac calcium (Ca^2+^) handling plays a fundamental role in the proper action potential (AP) generation and progression, contributing to the physiological contraction of the heart. Early studies have shown that hypertrophied myocytes present with a prolonged AP caused by changes in the ion channels’ expression and function, particularly Ca^2+^ related channels (McIntyre and Fry, 1997; Botchway et al., 2003). Hypertrophy also increases the predisposition for abnormal Ca^2+^ release events, the so-called spontaneous Ca^2+^ release from the sarcoplasmic reticulum (SR) (Brochet et al., 2012) by the ryanodine receptor (RyR) which provokes cytosolic Ca^2+^ overload. In LVH, this phenomenon is often counterbalanced by an enhanced expression of the sodium-Ca^2+^ exchanger (NCX). Additionally, the repolarization of hypertrophied myocytes is impaired due to a reduction in potassium currents. This triggers the generation of early or delayed afterdepolarizations (EADs or DADs) that in turn may lead to the re-opening of L-type Ca^2+^ channels (LTCCs), thus depolarizing the cell and inducing a premature ventricular beat (Tomita et al., 1994; Marionneau et al., 2008; Tse et al., 2016). Consequently, electrical waves may be initiated within the heart, predisposing cardiac tachyarrhythmias (Levy et al., 1987; Paar et al., 2019).

Besides disturbances in the cytosolic Ca^2+^ levels and fluxes, also mitochondrial Ca^2+^ handling has to be taken into consideration as it is a key regulator in diverse processes of cardiomyocyte physiology. First, mitochondria are the main providers of the energy needed for cardiac contraction via adenosine triphosphate (ATP). Increased mitochondrial Ca^2+^ cycling directly enhances the amount of ATP produced (Bers, 2002; Piquereau et al., 2013). Furthermore, mitochondria contribute in the modulation of the Ca^2+^ signalling within the cardiomyocyte, regulate the production of reactive oxygen species (Kohlhaas et al., 2010; Vakrou and Abraham, 2014), and may trigger cell death (Bernardi and Rasola, 2007; O'Rourke, 2007; Hoppe, 2010). The inner mitochondrial membrane is highly selective for Ca^2+^, which is tightly driven by the mitochondrial Ca^2+^ uniporter (MCU) complex, preventing the mitochondria from Ca^2+^ overload. The cardiac MCU complex is composed of the pore forming MCU protein (De Stefani et al., 2011) and multiple regulatory subunits, including the mitochondrial Ca^2+^ uptake 1 (MICU1) (Perocchi et al., 2010; Plovanich et al., 2013).

Additionally, besides the pore-forming subunits, several regulatory proteins determine the amount of Ca^2+^ that pass the inner mitochondrial membrane, such as protein arginine N-methyltransferase 1 (PRMT-1) and the mitochondrial uncoupling proteins (UCPs). PRMT-1 asymmetrically methylates MICU1 and reduces the sensitivity of MCU for Ca^2+^ (Madreiter-Sokolowski et al., 2016). In turn, the cardiac UCP-2 and UCP-3 isoforms normalize the inhibitory effects of PRMT-1 and re-activates the mitochondrial Ca^2+^ uptake (Trenker et al., 2007; Michels et al., 2009; Hoppe, 2010; Motloch et al., 2016a; Motloch et al., 2016b; Larbig et al., 2017; Paar et al., 2019).

Both pathologies, the acquired aortic valve stenosis (AVS) (Gunther and Grossman, 1979; Carabello and Paulus, 2009) and the congenital hypertrophic obstructive cardiomyopathy (HOCM) (Elliott et al., 2008; Elliott et al., 2014), may lead to a thickening of the left ventricle (LV) and concomitant cardiac remodelling on the tissue and cellular level. As both pathologies are known to promote cardiac arrhythmias, we hypothesized that cardiac remodelling might be the main cause for the development of an arrhythmogenic substrate. Of note, this accounts not only for the tissue level as in fibrosis, but also for changes on a molecular level. Regarding the crucial aspect of myocardial Ca^2+^ handling in cardiac arrhythmogenesis, we specially focussed on the expression of cytoplasmic and mitochondrial Ca^2+^ channels, as well as on the two most important SR-bound channels RyR2 and SR Ca^2+^-ATPase type 2a (SERCA2a). Due to the limited availability of human cardiac specimen, most of the previous studies were performed using animal models. To our knowledge, a molecular characterization of human myocardial cytoplasmic, mitochondrial, and SR Ca^2+^ channels and a direct comparison thereof in AVS and HOCM is still pending. Thus, we hypothesized, that both pathologies, AVS and HOCM, might be linked to major alterations in the expression of mitochondrial as well as cytosolic Ca^2+^ handling proteins. To investigate this issue, we analyzed septal specimen from cardiac surgery patients undergoing myectomy.

## 2 Methods

Patients suffering from severe, symptomatic AVS need to undergo aortic valve replacement to restore the functionality of the heart. As AVS may result in the asymmetric septal hypertrophy, the obstruction of the LVOT must be restored as well. Similarly, hypertrophic cardiomyopathy (HCM) may result in LVOT due do an excessive hypertrophy of the LV septum that obstructs the aortic blood flow, therefore termed HOCM. In order to restore physiological hemodynamics, part of this hypertrophic septal tissue is surgically removed in a process called myectomy (Kayalar et al., 2010; Hang et al., 2017). Thus, these specimens were further analysed in the present study.

### 2.1 Patient cohorts

The study was approved by the Austrian Ethics Committee of the State of Salzburg (415-E/2329/8-2018 and 415-E/2330/7-2018, respectively) and was conducted in accordance with the principles of the Declaration of Helsinki and Good Clinical Practice. Post-mortem, subvalvular LV septal specimen from six forensic autopsy cases served as healthy control (Ctrl) tissue. Specifically, only cases were included without reported cardiac diseases and without any evidence of macroscopically observed cardiac pathologies, such as cardiomyopathy or significant coronary artery disease during forensic autopsy. Furthermore, no individuals with macroscopically observed cardiac or major thoracic injuries (including damage from eventual resuscitation), as well as reported or diagnosed drug abused were used in the present study. In total, 21 subjects were enrolled into the study, comprised of six Ctrl subjects, eight AVS patients and seven HOCM patients. All participants from the AVS and HOCM groups signed a written informed consent prior to study participation. For all three study groups, the inclusion criteria were defined as age ≥18 years and for AVS and HOCM, a significantly asymmetrical LVH which results in LVOT requiring surgical removal. All included patients had no history of previous thoracotomy.

### 2.2 Assessment of clinical and echocardiographic parameters

A baseline echocardiographic examination was performed according to the ESC Guidelines for the management of cardiomyopathies (Ommen et al., 2020; Arbelo et al., 2023). These analyses included the functional assessment of the right- and left ventricle as well as evaluation of the valves prior to or at hospital admission and interpreted by a single cardiologist. The biplane Simpson method was used to calculate left ventricular ejection fraction (LVEF) and tricuspid annular plane systolic excursion (TAPSE) was used to delineate right ventricular function. Valvular heart disease was graded according to the 2021 ESC Valvular Heart Disease guidelines (Vahanian et al., 2022) using the baseline transthoracic and additional transesophageal echocardiographic data, if available. Important clinical data and the medical history of the patients was captured from in-hospital charts at the time of the surgical intervention.

### 2.3 Tissue processing

The septal cardiac samples were gained during cardiac surgery or in course of a forensic autopsy and were processed or fixed immediately after harvesting, respectively. Ctrl cardiac septal tissue was retrieved from the subvalvular left ventricular part of the IVS, within 24 h after death. Until forensic autopsy, the corpses were kept under cooled conditions in order to keep degradation to a minimum. Before further processing, the endocardium was removed to solely analyse the septal myocardium excluding the endocardial composition to avoid false increase in fibrotic burden. For histological examinations, the samples were either fixed in 4% formaldehyde solution (buffered, pH 6.9, Merck Millipore, MA, United States) overnight or an appropriate portion of the septal tissue was shock-frozen in liquid nitrogen and stored at −80°C for later immunoblotting.

### 2.4 Histological examination of formalin-fixed cardiac tissue

In order to determine potential tissue remodelling and the extent of fibrosis in hypertrophied septal specimen in comparison to healthy Ctrl tissue, the fixed tissue samples were dehydrated as described previously (Welsch et al., 2015). Then formalin-fixed paraffin-embedded (FFPE) tissue blocks were generated in Paraplast^®^ (Leica, Austria). Thereafter, the FFPE samples were cut into 6 µm thick sections and dried overnight. The next day, the tissue slices of all three study cohorts were stained by haematoxylin-eosin (HE), or Masson’s trichrome stain (Riedelsheimer and Büchl-Zimmermann, 2015), respectively. They were then investigated by two independent observers using an inverse microscope (Axiovert 200, Carl Zeiss, Germany) at a magnification of ×10 and 20x. Inflammatory infiltrates were detected and distinguished by size and shape of the blue-coloured cells in the HE-stained sections. The infiltrative foci were then graded according to semi-quantitative scales, which were previously reported (Eriksson et al., 2003; Aly et al., 2007). The grades were determined as follows: 0: no inflammatory infiltrates, 1: small foci of inflammatory cells between myocytes, 2: larger foci of inflammatory cells, 3: 10%–30% of a cross-section, 4: >30% of a cross-section (Eriksson et al., 2003; Aly et al., 2007; Mirna et al., 2021). For the quantification of the extent of fibrosis and the assessment of the cell sizes, a minimum of 15 pictures were taken by Axiocam MRc3 (Carl Zeiss, Germany) and AxioVision SE64 Rel. 4.8 (Carl Zeiss, Germany) to generate a highly random collection of tissue sections. The measurement of the cell sizes was performed in Zen 2.6 (blue edition; Carl Zeiss, Germany); the quantification of the blue (fibrotic) versus the red (cardiac muscle) areas was performed using ImageJ (NIH, LOCI, United States). The relative fibrotic area (RFA) was calculated as the pixels measured in blue versus red color, given as percentages.

In addition, Congo red staining was performed according to Riedelsheimer and Büchl-Zimmermann (Riedelsheimer and Büchl-Zimmermann, 2015) to histologically exclude amyloidosis of all study participants. Thereafter, the stained tissue sections were analyzed by light microcopy with a ×10 and ×20 objective (Carl Zeiss, Germany) and exemplary pictures were taken by Axiocam MRc3 (Carl Zeiss, Germany) and AxioVision SE64 Rel. 4.8 software (Carl Zeiss, Germany). In case of amyloidosis, Congo red binds to amyloid and red color would have been detected within the sections (Yakupova et al., 2019).

### 2.5 Protein studies by Western blot

The total protein content was extracted from the shock-frozen tissue sections by radio-immunoprecipitation assay (RIPA) lysis buffer (Santa Cruz Biotechnology, TX, United States) and the concentration was determined by a bicinchoninic acid (BCA) assay (Thermo Fisher Scientific, NH, United States). Thereafter, 10–30 µg of total protein was deployed for Western blot analysis of the fibrosis-relevant proteins, such as collagen 1 (COL1; abcam, Cambridge, United Kingdom), collagen 3 (COL3; abcam, Cambridge, United Kingdom), transforming growth factor-beta 1 (TGF-β1; abcam, Cambridge, United Kingdom), and mothers against decapentaplegic homolog 3 (SMAD3; Cell Signaling Technology, MA, United States). Equal amounts of protein lysates per protein of interest were separated by sodium dodecyl sulfate polyacrylamide gel electrophoresis (SDS-PAGE) on a 4%–15% Mini-PROTEAN TGX Gel (Bio-Rad Laboratories, Austria) with 4x Laemmli buffer (Bio-Rad Laboratories, Austria) serving as the loading buffer. Western blot bands were further transferred onto polyvinylidene fluoride (PVDF) Immun-Blot PVDF membranes (Bio-Rad Laboratories, Austria) by tank blotting. Membranes were blocked with an adequate blocking reagent, either EveryBlot Blocking Buffer (Bio-Rad Laboratories, Austria), ROTI®Block (Carl Roth, Germany), or 5% non-fat dry milk powder in TRIS-buffered saline with Tween-20 (TBS-T). Adequate blocking or diluting solutions were previously tested for each antibody. Membranes were incubated with the appropriate primary antibodies overnight at 4°C. The next day, after washing the membranes with TBS-T, the appropriate HRP-linked secondary antibodies (anti-rabbit or anti-mouse) were applied and incubated for 1 h at room temperature. The chemiluminescent signal was detected using Raytest Stella 8005 (Elysia-Raytest, Germany) and camera control software XStella Version 1.00.011 (Elysia-Raytest, Germany). In addition to the fibrosis-relevant proteins, Ca^2+^-associated ion channels and regulatory proteins were analyzed by Western blot. The analyses included: 1) the membrane-bound Ca^2+^channels: cardiac L-type Ca^2+^ channel (Ca_V_1.2; Alomone Labs, Jerusalem, Israel) and the Ca^2+^ extruding channel NCX1 (Cell Signaling Technology, MA, United States); 2) the two most important Ca^2+^ SR proteins RyR2 (abcam, Cambridge, United Kingdom) and SERCA2a (Santa Cruz Biotechnology, TX, United States); 3) the mitochondrial proteins MCU (Cell Signaling Technology), MICU1 (Cell Signaling Technology), UCP-2 (ProteinTech Europe, Manchester, United Kingdom), UCP-3 (abcam), and PRMT-1 (Cell Signaling Technology). The secondary anti-mouse and anti-rabbit IgG antibodies were obtained from Cell Signaling Technology. Glyceraldehyde-3-phosphate dehydrogenase (GAPDH; Cell Signaling Technology) and cytochrome c oxidase 4 (COX 4; abcam) were used as housekeeping protein for the normalization of the proteins of interest, as previously described elsewhere (Dally et al., 2006; Holmström et al., 2015; Danielsen et al., 2020; Zheng et al., 2022; Dridi et al., 2023). Data analysis and normalization were performed using Image Lab 6.0.1 software (Bio-Rad, Vienna, Austria). The bands were detected manually and the normalization channel and reference lane (references sample) were selected. Additionally, the background subtraction was set to a disk size of 70.0 mm. The normalized result of each sample was gained by automatic calculation, named “Norm- Vol. (Int).” Since the inclusion of the study participants was sporadic over a three-year period, as stated in the limitations section, the samples were run on different gels. To ensure comparability of the bands on different gels, an interblot control (stated as reference sample) was applied onto each individual gel, as previously reported elsewhere (Pillai-Kastoori et al., 2020; Taylor et al., 2022). After normalization of all samples with the housekeeping protein, we set the intensity of the reference sample to the value 1 (100%). Finally, we mathematically calculated the relative protein volume (RPV) by dividing the normalized sample of interest by the normalized reference sample, equally to the “IBC ratio” or “fold change” as also described in the literature (Pillai-Kastoori et al., 2020; Taylor et al., 2022). To create graphically representative and descriptive figures, the bands were graphically connected within the Western blot band figures (Figure 3A, 4A, and 5A).

### 2.6 Statistical analysis

Statistical analysis was performed using GraphPad PRISM 9 software (GraphPad-Software, San Diego, CA, United States). The distribution of the data was assessed by Shapiro-Wilk test. Non-parametric data are expressed as median and interquartile range (IQR; 25% to 75%-quartile), normally distributed data are given as mean and standard deviation (SD). Comparisons of two datasets were either performed using Welch’s test (unpaired) or paired *t*-test for normally distributed datasets. Non-parametric datasets were compared by Mann-Whitney test (unpaired) or Wilcoxon matched-pairs rank test (paired). A Welch’s ANOVA test for normally distributed data or a Kruskal–Wallis test for non-parametric data were used for the comparison of more than two datasets. These were split by the *post hoc* test Dunnett’s T3 multiple comparisons test (normally distributed) or Dunn’s multiple comparisons test (non-parametric), respectively. As some figures included both, normally-distributed and non-parametric datasets, all graphs of these figures are presented with median and interquartile range. To investigate the relation of the proteins analyzed with the severity of LVH or to draw a connection between the concentration of the different Ca^2+^-related proteins, either a Pearson correlation coefficient analysis (normally distributed data), or a non-parametric Spearman correlation were performed, respectively. The alpha threshold and the confidence level were set to *p* ≤ 0.05, which was determined as statistically significant.

## 3 Results

The analysis of all basic patient-related data showed a close similarity between the three study groups (see Table 1), with the exception of AVS: AVS patients displayed the highest age of all three study groups. Although ANOVA analysis of the ages of the three groups was significant (*p* = 0.0304), there was no significant difference between Ctrl vs. HOCM (*p* = 0.3036) or AVS vs. HOCM (*p* = 0.6063) groups, respectively.

**TABLE 1:** Basic study population characteristics of the Ctrl group, as well as the AVS and HOCM cohorts. Due to the normal distribution of the data, the values are given as mean ± SD.

Study population characteristics	Control (*n* = 6)	AVS (*n* = 8)	HOCM (*n* = 7)	*p*-value
Male	3/6	4/8	3/7	
Age (years)	46.3 ± 13.9	71.6 ± 6.8*	64.0 ± 16.5	0.0304
CAD	0/6	5/8	4/7	
Valvular disease	0/6	8/8	7/7	
COPD	0/6	0/8	1/7	
Arterial hypertension	1/6	8/8	5/7	
Hyperlipidemia	1/6	7/8	6/7	
Diabetes mellitus	1/6	3/8	0/7	

CAD: coronary artery disease; Ctrl: control; COPD: chronic obstructive pulmonary disease; *p < 0.05.

Furthermore, the major echocardiographic parameters as well as the clinical data, except from IVSd and AV Pmean, did not differ between the patients included in the AVS or HOCM study group, respectively (Table 2). Only a statistically significant elevation of IVSd in HOCM (*p* = 0.0204) in comparison to heathy Ctrl as well as a marked reduction in the mean atrial pressure in HOCM patients (*p* = 0.0302) was observed. No differences in right ventricular end-diastolic diameter were seen. In addition, none of the patients presented with typical echocardiography patterns suggesting cardiac amyloidosis, as previously described (Arbelo et al., 2023). This was also confirmed by Congo red stain of histological samples, shown in Supplementary Figure S1. Both AVS and HOCM presented as diffuse hypertrophy manifestations.

**TABLE 2:** Clinical data and echocardiographic parameters of the patients included in the AVS or HOCM study groups, respectively. Due to the normal distribution of the data, the values are given as mean ± SD.

Study population characteristics	AVS (*n* = 8)	HOCM (*n* = 7)	*p*-value
NYHA	2.0 ± 0.5	2.0 ± 0.5	>0.9999
Betablocker	6/8	6/7	
LVEF (%)	61.3 ± 10.3	64.6 ± 5.9	0.5040
IVSd (mm)	15.8 ± 3.1	22.7 ± 5.8*	0.0204
LVEDD (mm)	44.8 ± 9.1	36.9 ± 8.0	0.0959
LVESD (mm)	27.17 ± 7.2	20.9 ± 6.9	0.1280
TAPSE (mm)	20.6 ± 2.9	23.0 ± 4.6	0.2664
AV Pmax (mmHg)	88.0 ± 26.1	56.4 ± 39.46	0.1014
AV Pmean (mmHg)	52.88 ± 17.3	29.14 ± 19.8*	0.0302

AVP: atrial volume pressure; IVSd: interventricular septum thickness, diastolic; LVEDD: left ventricular end diastolic diameter; LVEF: left ventricular ejection fraction; LVESD: left ventricular end systolic diameter; mm: millimeters; mmHg: millimeters column of mercury; NYHA: new york heart association classification; SD: standard deviation; TAPSE: tricuspid annular plane systolic excursion; *p < 0.05.

### 3.1 Structural cardiac remodeling in LVH

**FIGURE 1:**
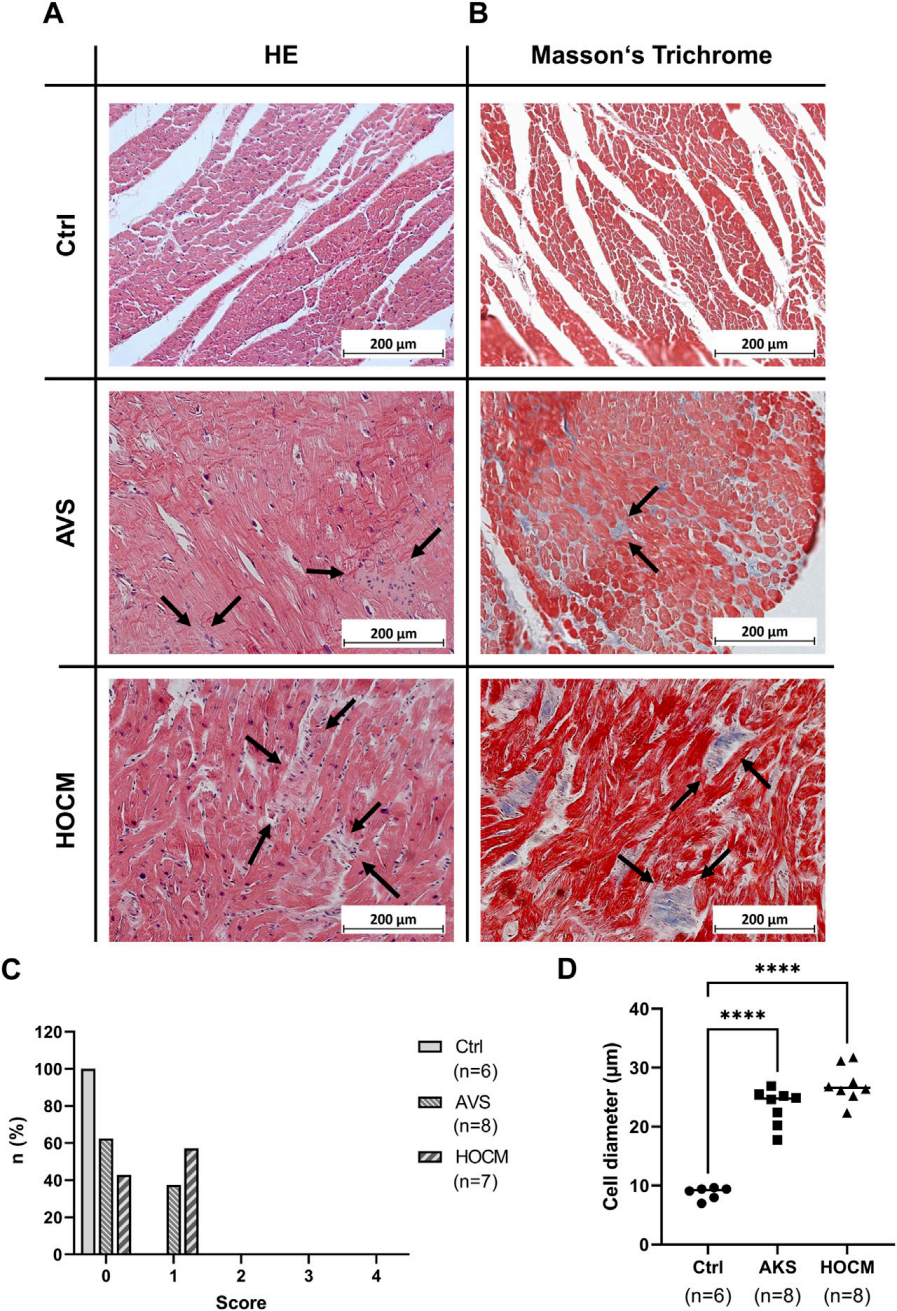
Light microscopy of cardiac tissue in Ctrl, AVS, and HOCM stained by HE **(A)** and Masson’s Trichrome **(B)**. In HE, cardiomyocytes appear dark-purple with large and round nucleus, while inflammatory infiltrates (indicated by arrows) appear much lighter and contain small sized nuclei. In Masson’s Trichrome, cardiomyocytes are stained in red, while fibrous tissue is stained in blue (indicated by arrows). **(C)** Histogram of the inflammatory regions (0: no inflammatory infiltrates, 1: small foci of inflammatory cells between myocytes, 2: larger foci of inflammatory cells, 3: 10%–30% of a cross-section, 4: >30% of a cross-section). While we could not detect any inflammatory focus in Ctrl (score 0), approximately 40% of AVS, and approximately 60% of HOCM exhibited inflammatory infiltrate score 1, respectively. **(D)** Histogram of the cardiomyocyte diameters in Ctrl vs. AVS/HOCM tissue. In diseased tissue, there was a substantial and statistically significant (****p < 0.0001) increase in cell diameter compared to Ctrl tissue.

Electrical signal transduction is highly dependent on the proper connection of two adjacent cardiomyocytes and an appropriate extent of extracellular matrix or connective tissue. In hypertrophy, several remodeling processes occur, with the increase in cardiomyocyte size as a primary sign. Furthermore, in acute cardiac diseases, inflammation is a major trigger for cardiac remodeling. Therefore, we investigated the extent of the cardiomyocyte growth in both diseases, as well as potential inflammatory infiltrates by standard histology (HE stain; Figure 1A). While we could not detect any inflammatory foci in any of the Ctrl samples (Score 0), the situation was slightly different in the pathological groups (depicted in Figure 1C): here 37.5% of the AVS (n = 8) and 57.1% of the HOCM sections analyzed had score 1 (n = 8). However, none of the samples analyzed had large foci of inflammatory infiltrates (score 3–4). When further analyzing cell sizes histologically (Figures 1A, B), the cardiomyocytes’ diameters reveal a substantial increase in the size of the hypertrophied cardiomyocytes in AVS as well as HOCM in comparison to the healthy Ctrl tissue (Figure 1D).

As cardiac hypertrophy is often accompanied by an extensive accumulation of fibrotic tissue (Vakrou and Abraham, 2014), the extent of fibrosis in the cardiac septum of healthy Ctrl individuals, AVS patients and HOCM patients was assessed histologically using Masson’s Trichrome. The qualitative examination of the microscopic pictures give rise to an increase of the fibrotic content (blue color) in AVS and the HOCM patients in comparison to the healthy Ctrl group (Figure 1B).

**FIGURE 2:**
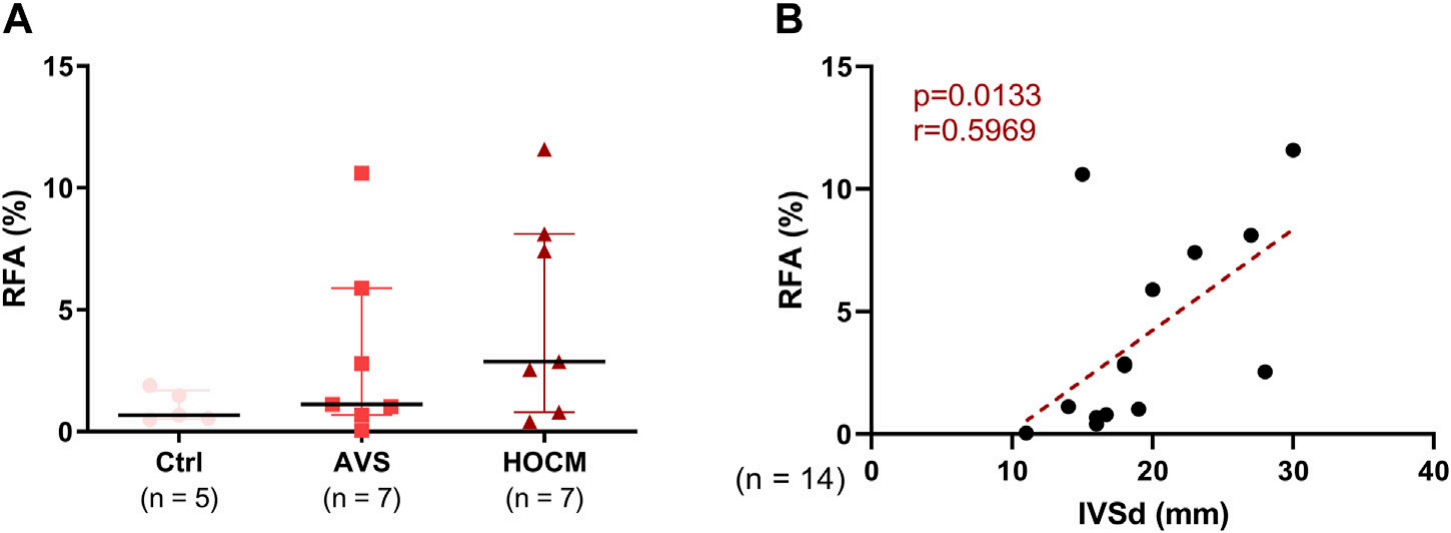
**(A)** Graphical comparison of the RFA of Ctrl, AVS, and HOCM tissue. The tissue sections were stained by Masson’s Trichrome stain and RFA was calculated by relating the resulting blue area (fibrous tissue) to the red area (cardiomyocytes). Due to non-Gaussian approximation, the data is depicted as median with IQR. **(B)** Relation of the RFA in both pathologies with the echocardiographically-measured diameter of the cardiac septum (IVSd) was obtained by Spearman correlation analysis. The dotted line depicts the regression curve of AVS and HOCM.

Quantitatively, the RFA was assessed as percentage between blue and red color in the whole section. Herein, the comparison of the RFA shows a slight trend towards an elevated fibrotic content in both pathologies, in comparison to the healthy Ctrl tissue (Figure 2A). Nevertheless, the RFA of all tissue analyzed strongly correlated with the echocardiographically measured IVSd. Confirming our assumptions, Figure 2B depicts a positive correlation of RFA with the IVSd (*p* = 0.0133) in both LVH pathologies, indicating that cardiac hypertrophy drives fibrotic accumulations.

**FIGURE 3:**
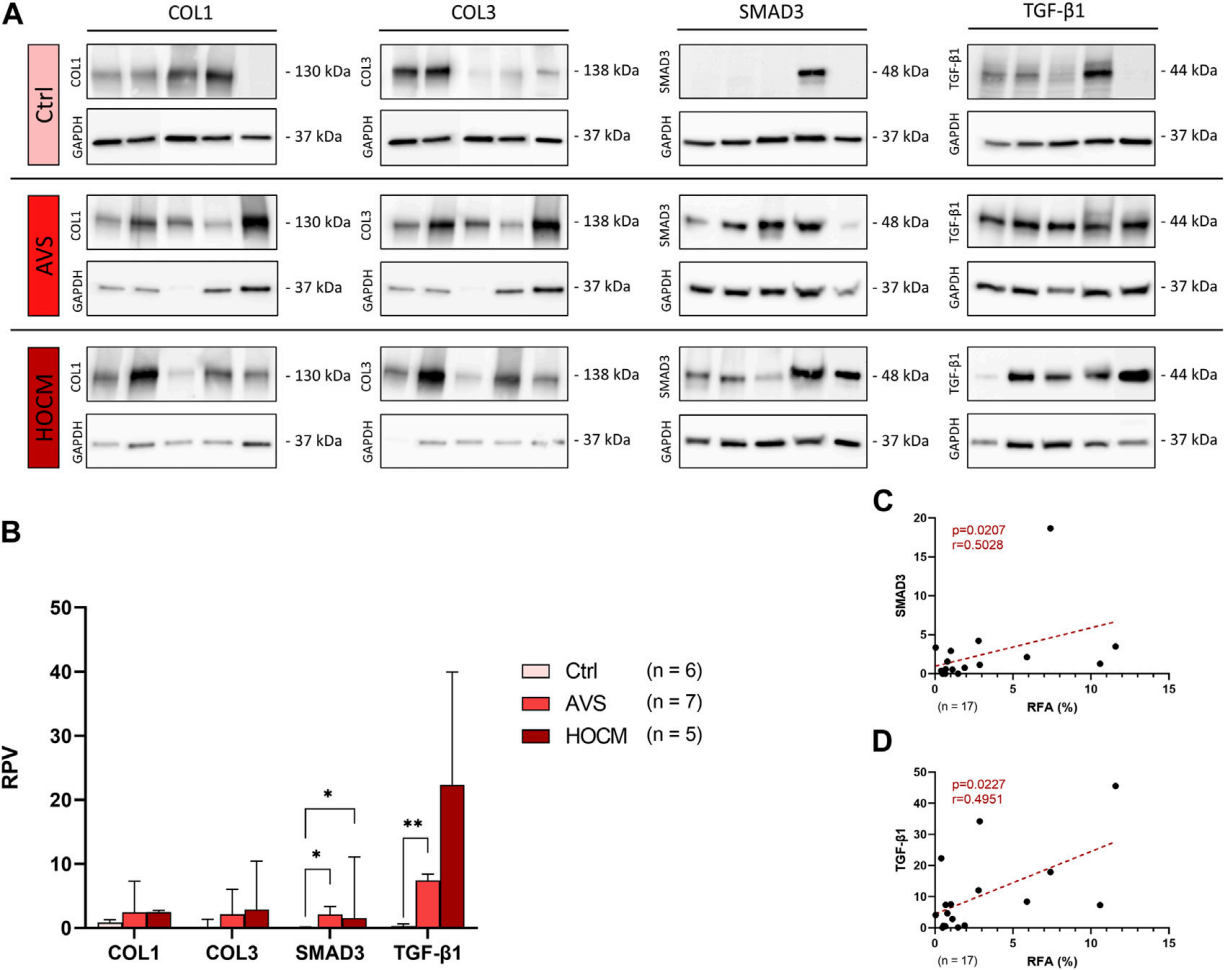
Western blot results of fibrosis-related proteins in Ctrl, AVS, and HOCM human cardiac tissue. RPV of each protein of interest is stated as a relative value to a unique reference sample. **(A)** Western blot bands of COL1, COL3, SMAD3, and TGF-β1 are normalized with GAPDH as housekeeping protein, respectively. Due to the study design, the depicted bands were graphically assembled from different blots. **(B)** Quantification of COL1, COL3, SMAD3, and TGF-β1 in human septal tissue, statistically analyzed by GraphPad PRISM. Due to non-Gaussian approximation of the figure’s data, RPVs are depicted as median and IQR with *p < 0.05 and **p < 0.01. **(C)** Spearman correlation of the RFA with the RPV of SMAD3 (n = 17). **(D)** Spearman correlation of the RFA with the RPV of TGF-β1 (n = 17). The dotted lines depict the regression curve of the Ctrl, AVS, and HOCM study groups.

In a further analysis, the quantification of the contents of fibrosis-related proteins in all three study groups was performed in relation to a unique reference sample of human septal tissue. As depicted in Figures 3A, B, the structural proteins COL1 and COL3 revealed a trend for an elevation in both diseases, though without statistical significance (*p* > 0.05). Nevertheless, SMAD3 increased markedly in AVS (*p* = 0.0180), as well as in HOCM (*p* = 0.0277) in comparison to the healthy Ctrl group. Additionally, RPV of TGF-β1 was significantly elevated in AVS (7.111 ± 2.982) in comparison to the Ctrl tissue (0.369 ± 0.312; *p* = 0.0028).

Accordingly, SMAD3 and TGF-β1 RPV values positively correlated with the RFA in both pathologies, as well as in the Ctrl tissue (Figures 3C, D). Evaluated by Spearman correlation, these data indicate that SMAD3 and TGF-β1 are linked with the fibrotic remodeling in LVH in both diseases.

### 3.2 Altered calcium channel expression in AVS and HOCM

**FIGURE 4:**
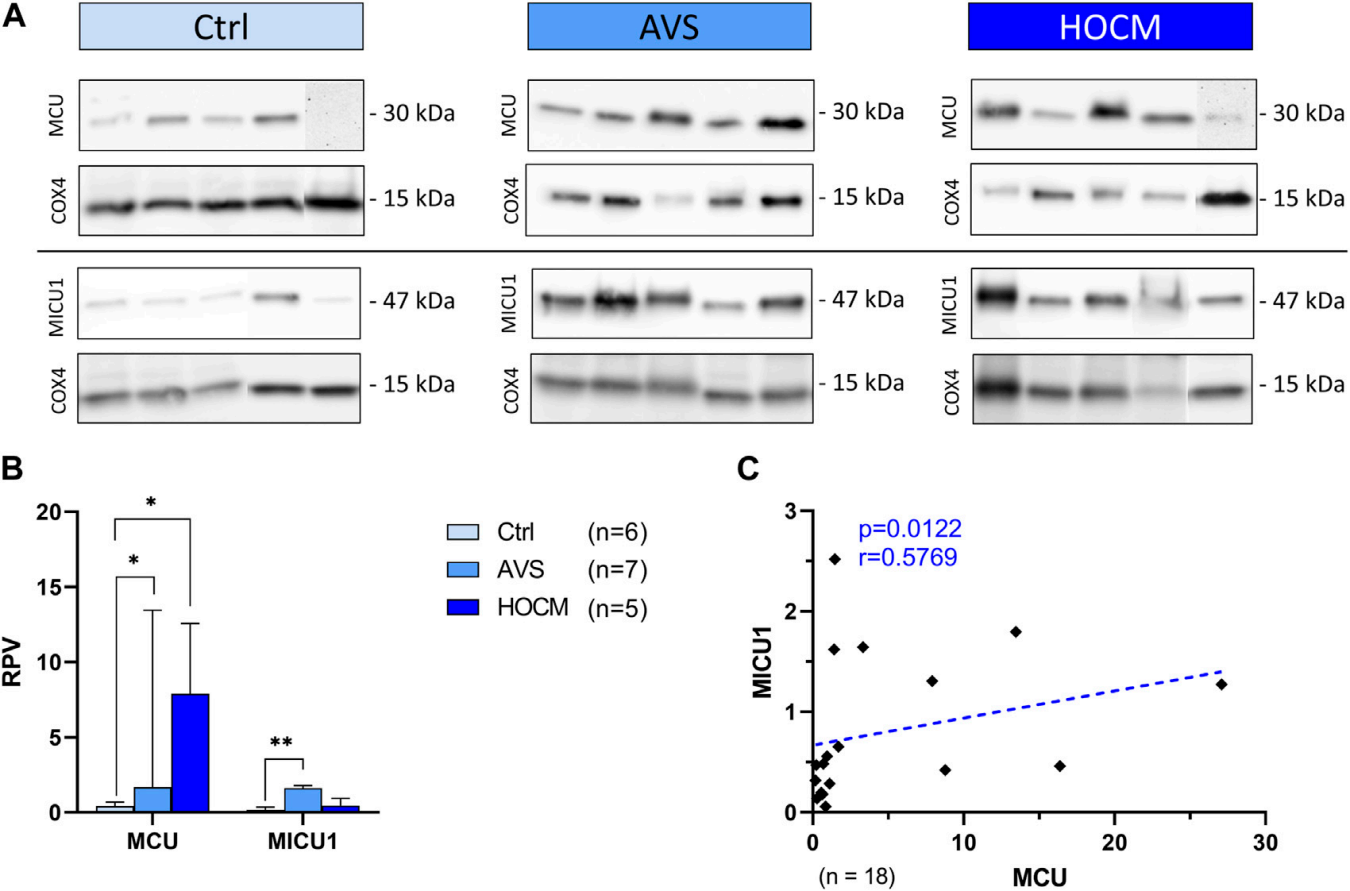
Western blot results of MCU complex-associated proteins in Ctrl, AVS, and HOCM human cardiac tissue. RPV of each protein of interest is stated as a relative value to a unique reference sample. **(A)** Western blot bands of MCU and MICU1 are normalized with COX4 as housekeeping protein. Due to the study design, the depicted bands were graphically assembled from different blots. **(B)** Quantification of MCU and MICU1 in human septal tissue. Due to non-normal distribution of MCU data, all data in the graph are given as median + IQR. The statistical significances were calculated using Kruskal–Wallis test for MCU and Welch ANOVA test for MICU1 with *p < 0.05 and **p < 0.01. **(C)** Spearman correlation of MCU with MICU1 of the whole study group. The dotted line depicts the regression curve.

In order to clarify the role of Ca^2+^ AVS and HOCM, as well as unveil potential alterations in the hypertrophied tissue, we performed Western blot analyses of the most important Ca^2+^ trafficking ion channels of cardiomyocytes. Most importantly, under physiological conditions Ca^2+^ trafficking between the cytosol and the inner mitochondrial membrane is tightly regulated. Changes in mitochondrial Ca^2+^ levels are mainly associated with alterations in the expression of the MCU complex proteins or its regulatory proteins. Therefore, we first focused on the presence and potential alterations of MCU and MICU1 proteins as the most important proteins building the functional MCU complex (Figures 4A–C). As shown in Figure 4B, MCU is highly increased in AVS (*p* = 0.0111), as well as in HOCM (*p* = 0.0172) in comparison to the Ctrl group. Similarly, LVH also increased the levels of MICU1 in AVS (*p* = 0.0084). However, no statistically significant alteration was observed between the two diseases, neither for MCU, nor for MICU1. Most importantly, there is a markedly positive relation between MCU and MICU1 expression patterns, indicating an overall increase in the density of the whole MCU complex (Figure 4C).

**FIGURE 5:**
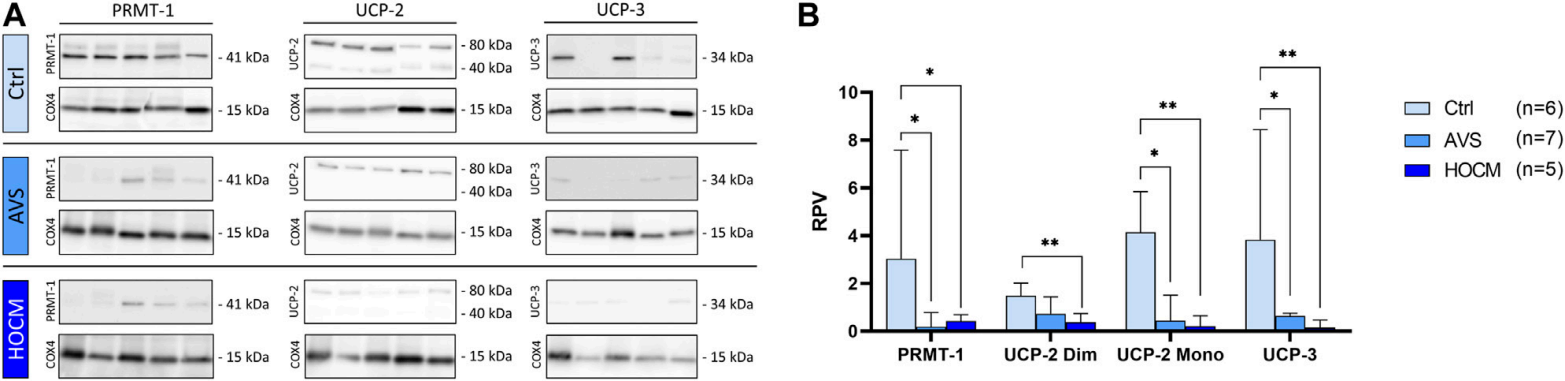
Western blot results of PRMT-1, UCP-2, and UCP-3 in Ctrl, AVS, and HOCM human cardiac tissue. RPV of each protein of interest is stated as a relative value to a unique reference sample. **(A)** Western blot bands of PRMT-1, UCP-2, and UCP-3 are normalized with COX4 as housekeeping protein. Due to the study design, the depicted bands were graphically assembled from different blots. **(B)** Quantification of PRMT-1, UCP-2, and UCP-3 in human septal tissue, statistically analyzed by GraphPad PRISM. Due to non-normal distribution, the statistical analysis was performed by Kruskal–Wallis test and the data is given as median + IQR with *p < 0.05 and **p < 0.01.

As MICU1 prevents mitochondria from Ca^2+^ overload by reducing the sensitivity of MCU for Ca^2+^ uptake in its methylated form, it may counteract the effects of an increase of MCU in the diseased tissue. However, MCU activity and MICU1 methylation are highly affected by their regulatory proteins UCP-2 and UCP-3, as well as PRMT-1. Therefore, the protein concentrations of all three proteins were assessed and quantified (Figure 5). As shown in Figure 5A, UCP-2 resulted in two separate bands, one specific to the monomeric UCP-2 protein, and the other appropriate to the dimeric form of UCP-2. In order to ensure that the UCP-2 results gained are valid, both UCP-2 bands were statistically analyzed separately. Therefore, the results of both bands are given separately as dimeric, called “Dim,” or monomeric, called “Mono.”

As seen in Figure 5B, PRMT-1 was greatly reduced in AVS (*p* = 0.0136), as well as in HOCM (*p* = 0.0400) in comparison to the Ctrl group. Functionally counteracting PRMT-1, the expression patterns of UCP-2 and UCP-3 were also significantly diminished in both diseases (AVS: *p* = 0.0485; HOCM: *p* = 0.0073) in comparison to the Ctrl group. Spearman correlation indicates the direct relation of the monomeric and dimeric UCP-2 bands (*p* < 0.0001, r = 0.8184; graph not shown).

**FIGURE 6:**
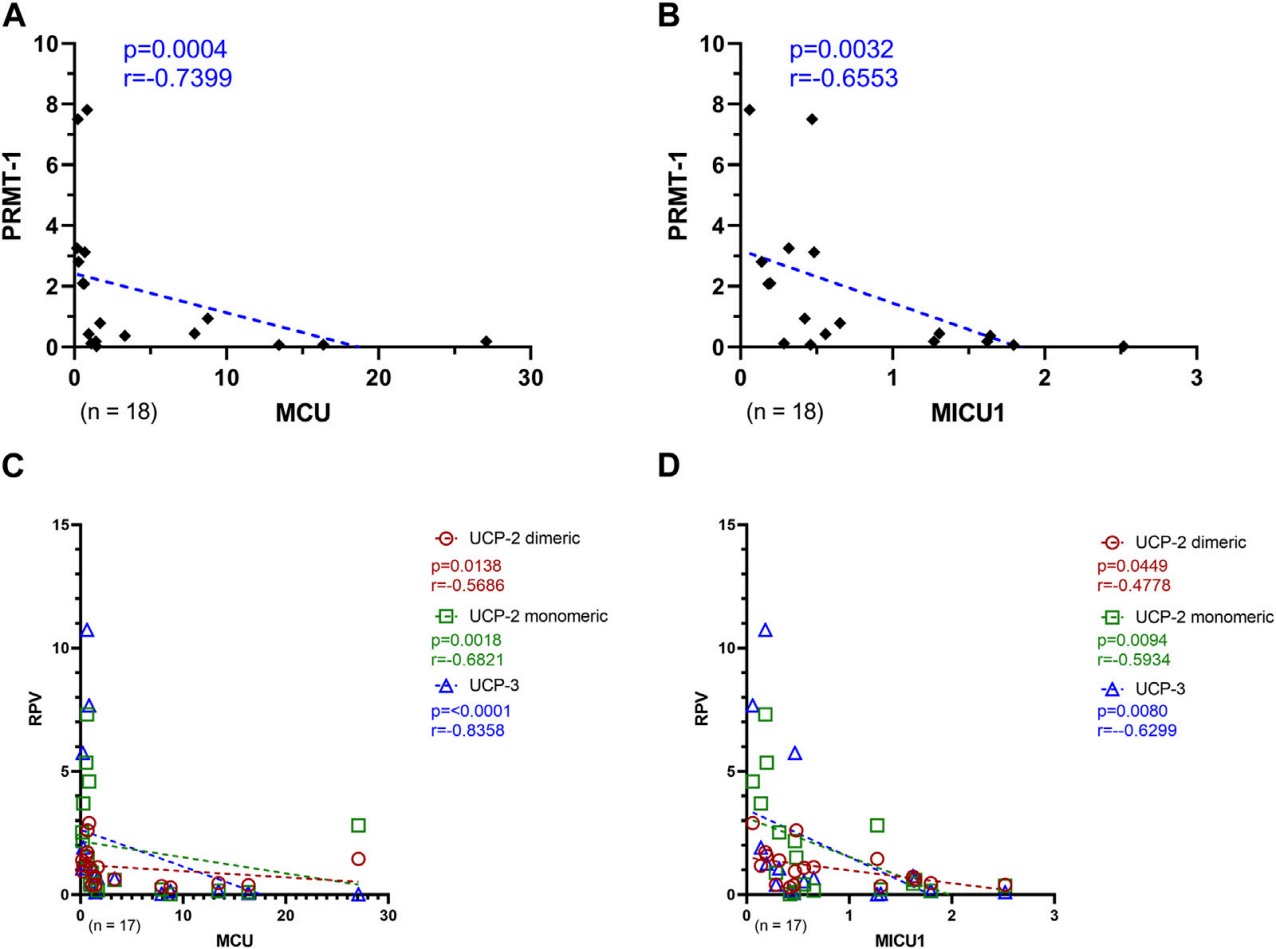
Relation of MCU complex regulatory proteins with MCU and MICU1 of Ctrl, AVS, and HOCM human cardiac tissue. **(A)** Spearman correlation of MCU with PRMT-1 including the whole study group. **(B)** Spearman correlation of MICU1 with PRMT-1. **(C)** Spearman correlation of MCU with the MCU or MICU1 regulatory proteins, UCP-2 Dim and Mono, as well as UCP-3, respectively. **(D)** Spearman correlation of MICU1 with the MCU or MICU1 regulatory proteins, UCP-2 Dim and Mono, as well as UCP-3, respectively. A *p*-value <0.05 is determined as statistically significant. The dotted lines depict the regression curve of all three study groups.

To investigate the relationship of the MCU complex regulatory proteins with MCU and MICU1, a Spearman correlation analysis of PRMT-1 with MCU, or MICU1 was performed, respectively. Additionally, the counterparts UCP-2 and UCP-3 were also put into relation with both MCU complex proteins. As depicted in Figures 6A, B, PRMT-1 negatively correlates with MCU and MICU1 expression patterns (MCU: *p* = 0.0004; r = -0.7399; MICU1: *p* = 0.0032, r = −0.6553). Furthermore, these findings are supported by the negative correlation of the RPV of the uncoupling proteins with MCU and MICU1 (Figures 6C, D).

**FIGURE 7:**
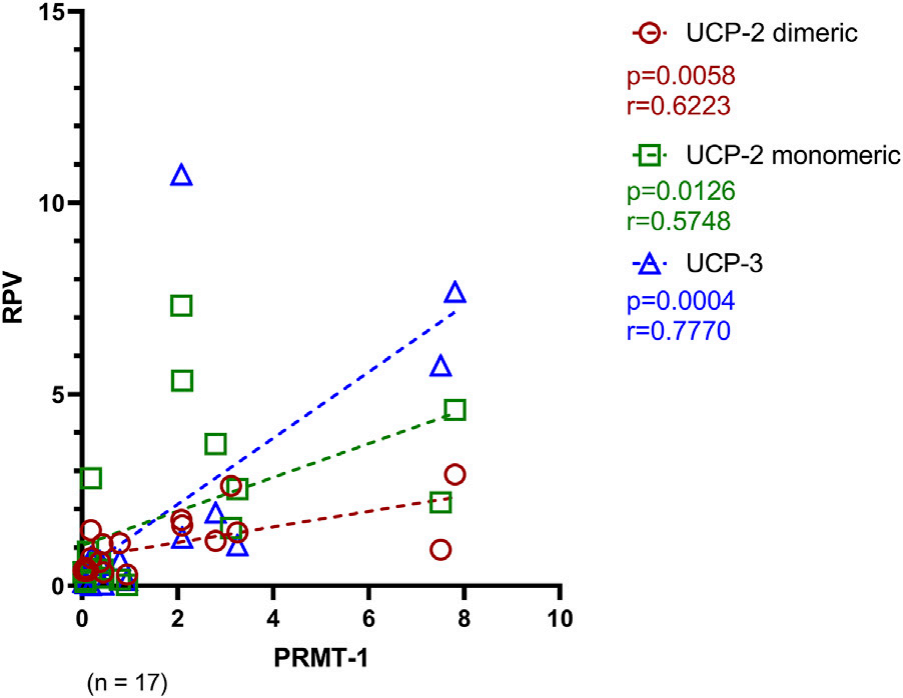
Spearman correlation of PRMT-1 with its counteracting proteins, UCP-2 and UCP-3 of Ctrl, AVS and HOCM. A *p*-value <0.05 is determined as statistically significant. The dotted lines depict the regression curve of all study groups.

PRMT-1, as the opponent of UCP-2 and UCP-3, decreases the sensitivity of MCU complex for Ca^2+^ trafficking. Therefore, to investigate a potential compensatory expression pattern of UCP-2 and UCP-3 with PRMT-1, a Spearman correlation analysis was performed. Most notably, as seen in Figure 7, there is a strong positive relation of the PRMT-1 concentration with both UCP-2 (dimeric: *p* = 0.0058; monomeric: *p* = 0.0126) and UCP-3 (*p* = 0.0004).

**FIGURE 8:**
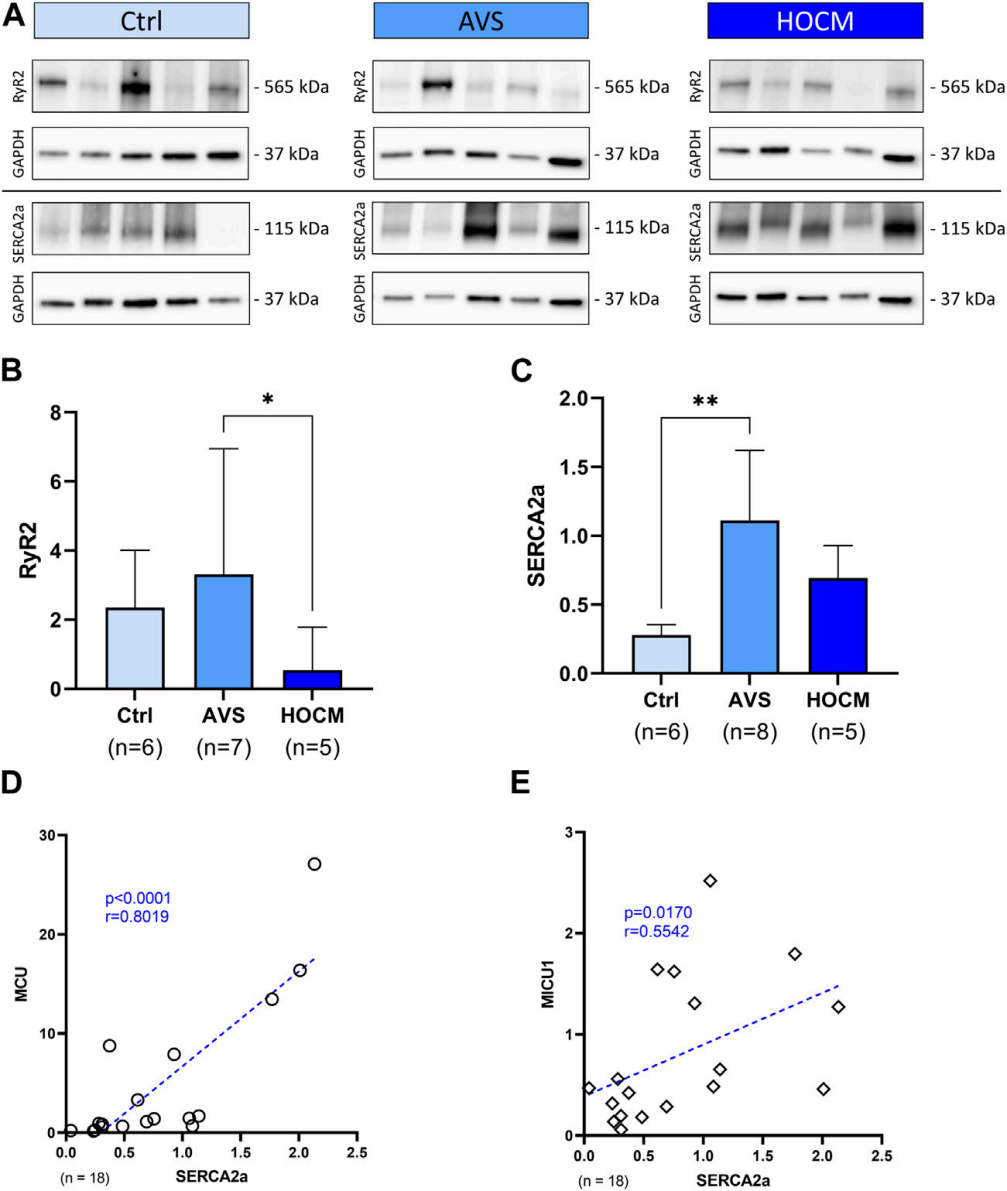
Western blot results of SR bound Ca^2+^-associated proteins in Ctrl, AVS, and HOCM human cardiac tissue. RPV of each protein of interest is stated as a relative value to a unique reference sample. **(A)** Western blot bands of RyR2 and SERCA2a are normalized with GAPDH as housekeeping protein. **(B)** Quantification of RyR2 in human septal tissue, statistically analyzed by GraphPad PRISM. Due to non-Gaussian approximation, the data was compared for their statistical differences using Kruskal–Wallis test and the bars are given as median + IQR with *p < 0.05 and **p < 0.01. **(C)** SERCA2a expression in human septal tissue. Due to normally distributed data, the statistical analysis was performed by Welch ANOVA test and the bars are given as mean + SD. **(D,E)** Spearman correlation of SERCA2a with MCU, or MICU1 Western blot results, respectively. The dotted line represents the regression line.

As mitochondrial Ca^2+^ uptake is highly dependent on a distinct Ca^2+^ threshold, mainly mediated by the SR, we also addressed the SR Ca^2+^ channels RyR2 and SERCA2a in our analyses (Figure 8). Our Western blot results and statistical analyses showed no significant difference between Ctrl and both pathology groups. Nevertheless, when comparing RyR2 in AVS (3.315; IQR: 6.948–1.744) and HOCM (0.539; IQR: 1.782–0.424) there was a reduction of RyR2 in HOCM (*p* = 0.0140), as seen in Figure 8A. In contrast, SERCA2a protein levels showed a significant elevation in AVS (*p* = 0.0013), though just a trend for an increased concentration in HOCM (Figure 8B). Furthermore, we found a statistically positive correlation between MCU and SERCA2a (r = 0.8019, *p* < 0.0001), or MICU1 (r = 0.5542, *p* = 0.0170), as depicted in Figures 8D, E.

**FIGURE 9:**
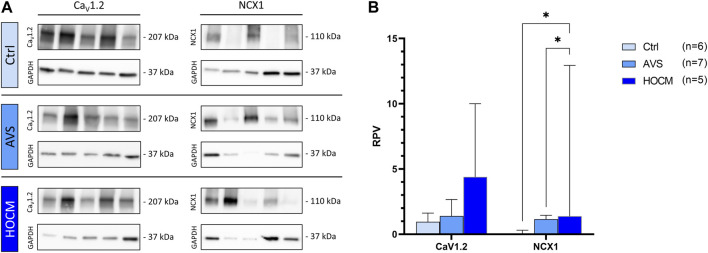
Western blot results of cell membrane-bound Ca^2+^-related channels in Ctrl, AVS, and HOCM human cardiac tissue. RPV of each protein of interest is stated as a relative value to a unique reference sample. **(A)** Western blot bands of CaV1.2 and NCX1 are normalized with GAPDH as housekeeping protein, respectively. Due to the study design, the depicted bands were graphically assembled from different blots. **(B)** Quantification of the cytoplasmic Ca^2+^ related ion channels Ca_V_1.2 and NCX1, statistically analyzed by GraphPad PRISM. Comparison of the channels’ RPV in AVS, HOCM, and healthy Ctrl septal tissue. Due to non-Gaussian approximation, the values in the graph are depicted as median + IQR with *p < 0.05.

In order to bring the cardiac Ca^2+^ handling into a full circle of Ca^2+^ trafficking ion channels, we also quantified the presence of the membrane-bound Ca^2+^ uptaking Ca_V_1.2 (the protein of the main cardiac LTCC) channel and *vice versa* the extruding ion pump NCX1. The results of the Western blot analyses of both proteins are depicted in Figure 9. Herein, we detected no significant alterations in Ca_V_1.2 levels in the hypertrophied myocardium in comparison to the Ctrl tissue (*p* > 0.05). On the other hand, NCX1 was highly elevated in both pathologies in comparison to the healthy Ctrl tissue (AVS: *p* = 0.0295; HOCM: *p* = 0.0449).

## 4 Discussion

Our study reveals structural and molecular remodeling characteristics in the human myocardium affected by AVS and HOCM, respectively, compared to healthy Ctrl tissue. Cardiac specimen was extracted from patients undergoing AV replacement with concomitant myectomy, due to a massive asymmetrical hypertrophy that obstructs the LVOT. Healthy Ctrl tissue was collected from forensic autopsy cases and equally processed and analyzed as AVS and HOCM tissue, respectively. Furthermore, to potentially unveil structural or molecular differences between both etiologies, we compared the outcomes of the acquired disease AVS with the congenital HOCM data.

Our analyses included 1) Basic patient characteristics and echocardiographic data, 2) investigations of structural alterations, such as hypertrophy, inflammation, and hypertrophy; and most importantly, 3) the assessment of potential alterations in cardiac Ca^2+^ ion channels.

Most of the patient characteristics did not differ between the study groups included. Both pathologies underlie a pronounced and diffuse LVH, clinically characterized by an increased IVSd and an obstruction of the LVOT. Nevertheless, based on the underlying etiologies, HOCM patients had a greater manifestation of hypertrophy in the septum than the AVS group. Additionally, as AVS arises in advanced ages (Nkomo et al., 2006), this study group tendentially represented the oldest group. Furthermore, we could clearly exclude additional manifestations of amyloidosis in all study cohorts by histological assessments and echocardiographic analysis of the AVS and HOCM study patients (Yakupova et al., 2019; Arbelo et al., 2023). Considering the clinical data gathered, and the fact that both pathologies are characterized by LV hypertrophy, we expected structural and molecular alterations in both pathologies. Most importantly, to our knowledge, the present study is the first that addresses potential alterations in the expression patterns of the MCU complex. Detailed combinational analyses of cell membrane bound and SR Ca^2+^ channels, particularly in AVS, are still speculative. Furthermore, a comparative analysis of the given aspects in AVS versus HOCM was never conducted to date.

The hypertrophic alterations in AVS and HOCM cardiac specimen were confirmed by the increase of the cardiomyocyte diameter in both pathologies. Cell enlargement of cardiomyocytes did not differ between AVS and HOCM. As far as the extracellular matrix is concerned, inflammatory infiltrates and the fibrotic burden were tendentially increased in LVH in comparison to healthy Ctrl tissue, although without statistical significance. Nevertheless, the fibrosis-promoting proteins TGF-β1 and SMAD3 were significantly elevated in LVH in the present study. As both proteins play crucial roles in the TGF-β pathway and their signaling triggers the accumulation of collagens (Vaughan et al., 2000; Lijnen et al., 2003; Khan et al., 2014; Khalil et al., 2017; Ma et al., 2018), we speculate that their elevation is associated with fibrotic remodeling processes in both diseases. Concomitant with the molecular findings, we found a positive correlation of the RFA and the IVSd, indicating a direct relation of LVH remodeling and the septum thickness in both diseases (Lúdvíksson and Gunnlaugsdóttir, 2003). These alterations found may profoundly trigger the propensity for malignant ventricular arrhythmias, which may lead to sudden cardiac death in their most severe manifestation (Varnava et al., 2000; Nazarian, 2011; Aro et al., 2017; Minners et al., 2020).

Besides alterations in the extracellular space, also remodeling processes in the context of cellular ion handling may tremendously influence the cardiac rhythmogenesis. Disturbances in cellular and mitochondrial Ca^2+^ handling have major impacts on the physiology of the cardiac APs, triggering early or delayed afterdepolarizations (Minners et al., 2020). Previous studies in animal models have already unveiled the relation of a disturbed contractile function of hypertrophied cardiomyocytes with a prolonged AP. As Ca^2+^ is a key responsible ion for the electrophysiologic behavior, alterations in its transporting channel expression and function therein may fundamentally influence the heart’s contractile function. From animal models of aortic banding, it is known that Ca^2+^ trafficking is increased in the compensatory stage of hypertrophy, which is mainly achieved by alterations in Ca^2+^ channel density and their increased sensitivity for Ca^2+^. However, these observations stemming from data from animal models cannot necessarily be directly applied to humans. Furthermore, none of the previous studies focused on mitochondrial Ca^2+^ trafficking in LVH due to AVS or HOCM (Sumida et al., 1998; Gattoni et al., 2017; Lewalle et al., 2018).

Severe cardiac hypertrophy is associated with Ca^2+^ overload, generating an arrhythmogenic substrate (McIntyre and Fry, 1997; Sipido et al., 2000; Botchway et al., 2003; Coppini et al., 2018). In our study, we addressed the expression patterns of the MCU complex-associated proteins MCU and its mediator MICU1 as predictors for the mitochondrial Ca^2+^ uptake phenotype. Interestingly, our analyses unveiled significant increases in MCU and MICU1 protein levels in AVS and HOCM, in comparison to the Ctrl tissue. MICU1 and MICU2 mainly act as gatekeepers that tightly regulate the amount of Ca^2+^ taken up by MCU (Plovanich et al., 2013; Kamer and Mootha, 2014; Patron et al., 2014). Especially in its unmethylated state and during high concentrations of Ca^2+^, MICU1 may activate the MCU pore to transport Ca^2+^ into the mitochondria (Patron et al., 2014; Garg et al., 2021). Supporting our findings, Paillard et al. (Paillard et al., 2022) found an altered MCU complex composition in the failing human heart. Although they could not reveal an increase in MCU contents, MICU1 and MICU2 levels were highly elevated with an increase in MICU1/MCU ratio (Paillard et al., 2022). In the present study, MICU1/MCU ratio dif not significantly differ between the study groups (*p* = 0.2223; data not shown), though there was a positive correlation between MICU1 and MCU levels.

Triggering MCU-mediated Ca^2+^ uptake, we also addressed the expression patterns of the MICU1 regulatory proteins PRMT1, UCP-2, and UCP-3. PRMT-1 methylates MICU1 and leads the suppression of Ca^2+^ uptake via the MCU pore (Madreiter-Sokolowski et al., 2016). As PRMT-1 as well as its counter players UCP-2 and UCP-3 were decreased in both pathologies, we assume that Ca^2+^ uptake by the mitochondria are positively triggered in LVH in comparison to healthy cardiomyocytes. Our suspicions are supported by two animal studies addressing MCU expression patterns in hypertrophic adaptions in response to pressure-overloaded murine hearts. Herein, microRNA-1 selectively targets the MCU protein and negatively affects its expression. On the other hand, the repression of microRNA-1 was shown to trigger cardiac hypertrophy, and *vice versa*, microRNA-1 is inhibited by the activation of β-adrenoreceptors in hypertrophy, leading to the MCU translation (Zaglia et al., 2017). Finally, Yu et al. (Yu et al., 2018) showed a direct link of MCU density with ventricular enlargement together with a ventricular asynchrony in pressure-overloaded heart failure. In contrast, most recent studies indicate a positive effect of MCU upregulation in hypertrophy and heart failure. Since the heart samples in the present study represent rather a moderate, though not an end-stage, hypertrophy or heart failure, the increase in MCU expression found may indicate a compensatory stage of hypertrophy. Thus, the presence of elevated MCU may counteract SR Ca^2+^ leaks and potentially restore mitochondrial Ca^2+^ homeostasis (Hamilton et al., 2021; Liu et al., 2021). Nevertheless, since our study did not measure Ca^2+^-uptake this speculation need to be interpreted with caution. Consequently, MCU expression or function may indicate a potential target for future therapeutic interventions in AVS, or HOCM, respectively.

In addition to aforementioned marked alterations in MCU complex-associated proteins, hypertrophy leads also to a disarrangement of the transverse-tubules (t-tubules) and the SR, increasing the space between LTCC and the RyR2 of the SR. Consequently, the number of uncoupled RyR2 increase, resulting in a reduced or delayed CICR from the SR. This directly influences the synchrony of Ca^2+^ release, which alters the Ca^2+^ transient amplitude of the AP and has a direct effect on Ca^2+^-dependent arrhythmias (Hamilton and Terentyev, 2019). In our study, we found RyR2 to be reduced in HOCM in comparison to AVS. However, no statistically significant alteration was found in comparison to healthy Ctrl. We might cautiously interpret the slight (though without significant difference) increase of RyR2 in AVS by the previously reported need of RyR2 for compensated hypertrophy in response to pressure overload (Zou et al., 2011). More recently, an important study by Zheng et al. (Zheng et al., 2022) reported that RyR2 deficiency clearly affects Ca^2+^ sparks and amplitudes with mild desynchronization of the Ca^2+^ transients. Nevertheless, the functional performance and the global Ca^2+^ release is fully compensated (Zheng et al., 2022). Therefore, we suggest that the slight reduction in RyR2 in HOCM patients would not markedly affect the Ca^2+^ related excitation-contraction coupling.

As functional counter player of RyR2, we found SERCA2a to be significantly elevated in AVS in the present study in comparison to Ctrl. Supporting our present findings, an increase in SERCA2a expression is reported in the compensatory stage of hypertrophy during which the myocardial function and the contractile force is preserved (Ding et al., 2000; Ito et al., 2000; Silva et al., 2022). In this context, an improved Ca^2+^ trafficking in the SR, mediated by changes in expression levels or its regulating protein phospholamban, may be beneficial in hypertrophic or failing cardiomyocytes. However, the affinity of SERCA2a for Ca^2+^ has to be tightly regulated since it plays a prominent role in the progression of hypertrophy and cardiac dysfunction (Vangheluwe et al., 2006).

To further evaluate the interplay of cardiac Ca^2+^ channels, we finally addressed the expression levels of the membrane bound Ca^2+^ uptaking channel Ca_V_1.2 (LTCC) and the extruding ion pump NCX1. Although without statistical significance, Ca_V_1.2 levels were slightly increased in both pathologies. Most importantly, Coppini et al. (Coppini et al., 2018) found a cytosolic Ca^2+^ overload in HCM cardiomyocytes due to an increased trafficking of Ca^2+^ though LTCC. This was most recently confirmed by Medvedev et al. (2021) demonstrating a higher probability for open LTCCs in failing cardiomyocytes. In contrast, NXC1 is responsible for the extrusion of cytosolic Ca^2+^ into the extracellular space. Our data from AVS and HOCM patients confirm previous studies that found an upregulation of NCX1 in the hypertrophic or failing heart (Wang et al., 2001; Müller et al., 2002). This increase in expression may be associated with a decline in contractility and lead to abnormal Ca^2+^ transients, further promoting the propensity for arrhythmias (Wang et al., 2009; Ujihara et al., 2016). Nevertheless, since Wang et al. excluded the onset of heart failure 3–5 weeks after NCX1 transgene overexpression (Wang et al., 2009), we conclude that the present increase of NCX1 result from the LVH in AVS or HOCM, respectively, rather than being responsible for the progression of both pathologies (Menick et al., 2013).

## 5 Conclusion

Taken together, our findings confirm structural changes in the hypertrophied myocardium in AVS and HOCM. In line with earlier studies, these alterations may be associated with changes in the electrophysiological network of the heart. In the present study, there is a direct connection between the fibrotic burden and the grade of septal hypertrophy. Additionally, as shown by the analysis of the Ca^2+^ related ion channels of cardiomyocytes, several alterations of the cellular and mitochondrial Ca^2+^ take place. Most notably, we clearly demonstrated alterations in the levels of MCU complex associated proteins that indicate disturbances of the mitochondrial Ca^2+^ balance. Finally, our study shows that the alterations found in either AVS, or HOCM, were statistically even in both pathologies. To our knowledge, we unveiled significant structural changes concerning Ca^2+^ ion channels in LVH due to AVS, or HOCM in humans for the first time, respectively. Additionally, this is the first study that directly compares those structural changes in the acquired disease AVS with the congenial etiology of HOCM. Our study also provides first evidence for potential therapeutic targets in patients suffering from cardiomyopathies in LVH, triggered by AVS or HOCM, respectively.

## 6 Limitations

The present study greatly supports and confirms previous studies performed in animal models. Of note, all individuals included into the Ctrl cohort were markedly younger than the patients enrolled in the AVS or HOCM groups. Furthermore, compared to the control group, patients in the AVS and HOCM group were affected by CADs, which might promote a higher fibrotic burden in both pathologies. As healthy cardiac tissue is only gained under special circumstances, such as death due to other (impaired health) reasons than cardiovascular diseases, we were restricted to specimens gained from post-mortem individuals. Therefore, we could not eliminate age-associated differences in the cardiac tissue analyzed. Although septal tissue was removed by myectomy in LVH, human cardiac tissue is not abundantly available. While immunohistochemistry was not performed, we were unable to discriminate between activated or non-activated endothelial cells in our HE-sections, while inflammatory cells were discernible by size and shape. Therefore, it seems unlikely that this issue would significantly alter our results. Additionally, an overall greater number of inclusions in each study cohort may have statistically confirmed the expected significant increase in the fibrotic burden in LVH in both diseases. As the study was designed and performed during the COVID-19 pandemic, cardiac surgery interventions were postponed or the patients were simply not included due to the additional efforts during that time. Consequently, inclusion numbers were reduced and patients not included in a timely or steady manner. Whether the alterations in the mitochondrial and SR Ca^2+^ related proteins have direct functional or cytotoxic effects on the contraction property or the electrophysiological properties of the diseased heart remains to be elucidated in future studies. As the isolation of human ventricular cardiomyocytes from small portions of cardiac tissue is rather difficult, we still failed to perform functional analyses, as well as electrophysiological examinations of the cellular and mitochondrial Ca^2+^ fluxes. Together with the clinical data, these analyses would deepen the understanding of the underlying pathophysiologies. This concern should be investigated in further trials.

## Figures and Tables

**Table:** 

**AP**	Action potential
**ATP**	Adenosine triphosphate
**AVS**	Aortic valve stenosis
**AV P**	Atrial volume pressure
**BCA**	Bicinchoninic acid
**Ca2+**	Calcium
**CAD**	Coronary artery disease
**COL**	Collagen
**COPD**	Chronic obstructive pulmonary disease
**COX4**	Cytochrome c oxidase 4
**Ctrl**	Control
**DAD**	Delayed afterdepolarization
**EAD**	Early afterdepolarization
**FFPE**	Formalin-fixed paraffin-embedded
**GAPDH**	Glyceraldehyde-3-phosphate dehydrogenase
**HCM**	Hypertrophic cardiomyopathy
**HE**	Haematoxylin-eosin
**HOCM**	Hypertrophic obstructive cardiomyopathy
**IQR**	Interquartile range
**IVSd**	Interventricular septum thickness, diastolic
**LTCC**	L-type calcium channel
**LV**	Left ventricle
**LVEDD**	Left ventricular end diastolic diameter
**LVESD**	Left ventricular end systolic diameter
**LVEF**	Left ventricular ejection fraction
**LVH**	Left ventricular hypertrophy
**LVOT**	Left ventricular outflow tract
**MCU**	Mitochondrial calcium uniporter
**MICU1**	Mitochondrial calcium uptake 1
**NCX**	Sodium-calcium exchanger
**NYHA**	New York Heart Association classification
**PRMT-1**	Protein arginine N-methyltransferase 1RFA Relative fibrotic area
**RPV**	Relative protein volume
**RIPA**	Radio-immunoprecipitation assay
**RyR**	Ryanodine receptor
**SD**	Standard deviation
**SDS-PAGE**	Sodium dodecyl sulfate polyacrylamide gel electrophoresis
**SERCA2a**	Sarcoplasmic reticulum calcium- adenosine triphosphatase type 2a
**SMAD3**	Mothers against decapentaplegic homolog 3
**SR**	Sarcoplasmic reticulum
**TAPSE**	Tricuspid annular plane systolic excursion
**TGF-β1**	Transforming growth factor-beta 1
**T-tubules**	Transverse tubules
**UCP**	Mitochondrial uncoupling protein

## Data Availability

The original contributions presented in the study are included in the article/Supplementary Material, further inquiries can be directed to the corresponding author.

## Supplementary material

SUPPLEMENTARY FIGURE S1 Histological samples of Ctrl, AVS, and HOCM patients, stained by Congo red in order to exclude amyloidosis. The cell nuclei were stained in blue. As amyloid was not detected, no red color is visible. Microscopic pictures were taken by a ×20 objective.
