# Supplementary figures and images for: Hypertrophic cardiomyopathy is characterized by alterations of the mitochondrial calcium uniporter complex proteins: insights from patients with aortic valve stenosis versus hypertrophic obstructive cardiomyopathy
Source: Front Pharmacol. 2023 Nov 22;14:1264216. doi: 10.3389/fphar.2023.1264216 (PMC10703305; doi:10.3389/fphar.2023.1264216)

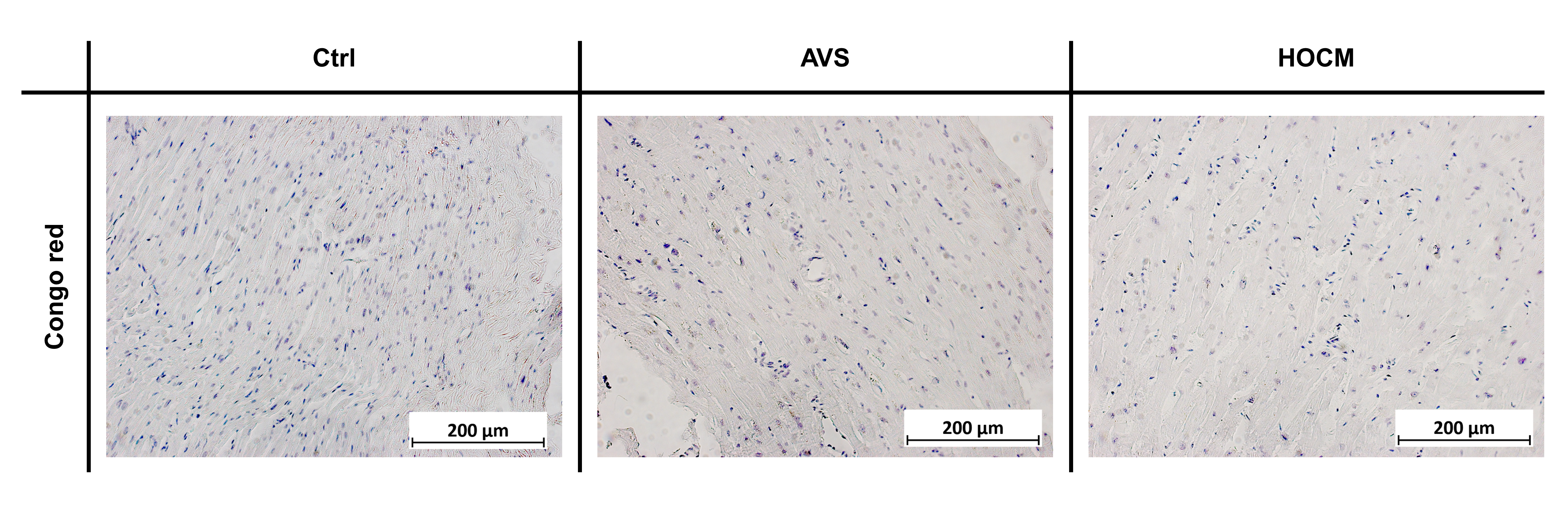

Supplement: Supplementary file 1 [file Image1.TIF]
